# Drying banana seeds for *ex situ* conservation

**DOI:** 10.1093/conphys/coab099

**Published:** 2022-01-12

**Authors:** Simon Kallow, Manuela Garcia Zuluaga, Natalia Fanega Sleziak, Bayu Nugraha, Arne Mertens, Steven B Janssens, Lavernee Gueco, Michelle Lyka Valle-Descalsota, Tuong Dang Vu, Dang Toan Vu, Loan Thi Li, Filip Vandelook, John B Dickie, Pieter Verboven, Rony Swennen, Bart Panis

**Affiliations:** Royal Botanic Gardens Kew, Millennium Seed Bank, Wakehurst, Ardingly, Sussex, RH17 6TN, UK; Department of Biosystems, Katholieke Universiteit Leuven, Willem de Croylaan 42, 3001 Leuven, Belgium; Meise Botanic Garden, Nieuwelaan 38, 1860 Meise, Belgium; Department of Biosystems, Katholieke Universiteit Leuven, Willem de Croylaan 42, 3001 Leuven, Belgium; Department of Biosystems, Katholieke Universiteit Leuven, Willem de Croylaan 42, 3001 Leuven, Belgium; Department of Biosystems, Katholieke Universiteit Leuven, Willem de Croylaan 42, 3001 Leuven, Belgium; Agricultural and Biosystems Engineering Department, Universitas Gadjah Mada, Jl. Flora No. 1, Sleman, Yogyakarta 55281, Indonesia; Department of Biosystems, Katholieke Universiteit Leuven, Willem de Croylaan 42, 3001 Leuven, Belgium; Meise Botanic Garden, Nieuwelaan 38, 1860 Meise, Belgium; Meise Botanic Garden, Nieuwelaan 38, 1860 Meise, Belgium; Department of Biology, Katholieke Universiteit Leuven, Kasteelpark Arenberg 31, 3001 Leuven, Belgium; National Plant Genetic Resources Laboratory, Institute of Plant Breeding, College of Agriculture and Food Science, University of the Philippines, Los Baños, 4031 Laguna, Philippines; National Plant Genetic Resources Laboratory, Institute of Plant Breeding, College of Agriculture and Food Science, University of the Philippines, Los Baños, 4031 Laguna, Philippines; Research Planning and International Cooperation Department, Plant Resources Center, VAAS, Ha Noi, Viet Nam; Research Planning and International Cooperation Department, Plant Resources Center, VAAS, Ha Noi, Viet Nam; Genebank Management Division, Plant Resources Center, VAAS, Ha Noi, Viet Nam; Meise Botanic Garden, Nieuwelaan 38, 1860 Meise, Belgium; Royal Botanic Gardens Kew, Millennium Seed Bank, Wakehurst, Ardingly, Sussex, RH17 6TN, UK; Department of Biosystems, Katholieke Universiteit Leuven, Willem de Croylaan 42, 3001 Leuven, Belgium; Department of Biosystems, Katholieke Universiteit Leuven, Willem de Croylaan 42, 3001 Leuven, Belgium; International Institute of Tropical Agriculture, Plot 15B Naguru East Road, Upper Naguru, Box 7878, Kampala, Uganda; Department of Biosystems, Katholieke Universiteit Leuven, Willem de Croylaan 42, 3001 Leuven, Belgium; Bioversity International, Willem de Croylaan 42, 3001 Leuven, Belgium

**Keywords:** Crop wild relatives, desiccation tolerance, genetic resources, genebank

## Abstract

The ability of seeds to withstand drying is fundamental to *ex situ* seed conservation but drying responses are not well known for most wild species including crop wild relatives. We look at drying responses of seeds of *Musa acuminata* and *Musa balbisiana*, the two primary wild relatives of bananas and plantains, using the following four experimental approaches: (i) We equilibrated seeds to a range of relative humidity (RH) levels using non-saturated lithium chloride solutions and subsequently measured moisture content (MC) and viability. At each humidity level we tested viability using embryo rescue (ER), tetrazolium chloride staining and germination in an incubator. We found that seed viability was not reduced when seeds were dried to 4% equilibrium relative humidity (eRH; equating to 2.5% MC). (ii) We assessed viability of mature and less mature seeds using ER and germination in the soil and tested responses to drying. Findings showed that seeds must be fully mature to germinate and immature seeds had negligible viability. (iii) We dried seeds extracted from ripe/unripe fruit to 35–40% eRH at different rates and tested viability with germination tests in the soil. Seeds from unripe fruit lost viability when dried and especially when dried faster; seeds from ripe fruit only lost viability when fast dried. (iv) Finally, we dried and re-imbibed mature and less mature seeds and measured embryo shrinkage and volume change using X-ray computer tomography. Embryos of less mature seeds shrank significantly when dried to 15% eRH from 0.468 to 0.262 mm^3^, but embryos of mature seeds did not. Based on our results, mature seeds from ripe fruit are desiccation tolerant to moisture levels required for seed genebanking but embryos from immature seeds are mechanistically less able to withstand desiccation, especially when water potential gradients are high.

## Teaser

We investigated responses to drying for seeds of banana wild relatives to improve seed conservation for food security. We found that mature seeds were desiccation tolerant to levels required for seed genebanking but less mature seeds and those from unripe fruit that may be inadvertently collected have low viability and are sensitive to fast desiccation.

## Introduction

The aim of seed conservation is to store the maximum amount of plant genetic diversity for prolonged periods of time and make it accessible for future uses. Banked seeds may then be employed for restoration, assisted migration, research or, for crops and crop wild relatives (CWRs), breeding (UN General Assembly, 2015; Peres, 2016). Conserving banana and plantain (referred to as ‘banana’) genetic resources is crucial because banana crops are one of the world’s most important (FAO, 2020a; FAO, 2020b); however, production is threatened by a host of diseases (Boloy *et al.,* 2014; Ploetz and Evans, 2015; Jones, 2018; Fones *et al.,* 2020), as well as climate change (Ramirez *et al*., 2011; Varma and Bebber, 2019). Furthermore, of 59 banana CWR species recently assessed for conservation, 9 were endangered, 11 were threatened with extinction and 56 were insufficiently conserved *ex situ*, including in seed genebanks (Mertens *et al.,* 2021). Banana seed conservation is therefore a global priority for food security (Kallow *et al.,* 2021b; Castañeda-Álvarez *et al.,* 2016).

The two key components of seed genebanking of orthodox seeds are drying and cooling (FAO, 2014). Drying orthodox seeds in combination with reducing storage temperature increases longevity in storage (Harrington, 1972; Ellis and Roberts, 1980; Vertucci and Roos, 1990). Orthodox seeds are, by definition, desiccation tolerant: they can be dried to ≤3–7% fresh weight moisture content (MC) and can be stored at sub-zero temperatures without loss of viability (Roberts, 1973). Whereas most plant species produce orthodox seeds (Wyse and Dickie, 2017), many produce recalcitrant seeds (Berjak and Pammenter, 2008). Drying recalcitrant seeds to below ~10% MC kills them. Longevity of recalcitrant seeds cannot be extended by drying; the application of advanced cryogenic protocols may be required for their storage (Walters *et al*., 2013). Alternatively, other species produce seeds that are somewhat in-between orthodox and recalcitrant storage class and are referred to as intermediate. They show partial sensitivity to drying and storage at sub-zero temperatures, so conventional storage is also not an option for these species (Ellis *et al*., 1990). Characterizing seed responses to drying is therefore essential to applications of seed conservation.

While seeds of different species broadly fit into these categories, there is inevitably a degree of variation in desiccation response between and within seed batches of the same taxa. For instance, post-storage viability is usually represented as a percentage, meaning it is expected that within a batch some seeds survive storage and remain viable and some seeds do not (Leprince, 2004). Seed maturity is a primary factor influencing desiccation tolerance and seed longevity, and this may vary in a seed batch (Hay and Smith, 2004). As seeds mature, their morphology and biochemistry changes, influencing desiccation responses and longevity in storage (Leprince *et al*., 2017; Ellis, 2019). After fertilization, seed filling occurs until maximum dry weight is achieved. Seeds are then at mass maturity, sometimes known as physiological maturity. At this point abscission from the mother plant occurs and no further nutrients are therefore received. Following mass maturity, for dry-dispersed seeds, and to lesser extent seeds of fleshy fruits (like bananas) (Demir and Ellis, 1992a; Demir and Ellis, 1992b), a period of late maturation occurs, during which further moisture is lost and seeds equilibrate close to ambient relative humidity (RH). This stage is important to protect cell structure and avoid lethal shrinkage during desiccation (Walters, 2015; Leprince *et al.,* 2017; Ballesteros *et al*., 2020).

In addition to maturity level, maternal provenance and environment impact seed desiccation tolerance levels and longevity (Ellis *et al.*, 1991; Daws *et al.,* 2006; Berjak and Pammenter, 2008). Seed anatomy such as the thickness and permeability of the testa and the chemical content of cells also influence how water potential gradients are regulated in seeds, and thus how seeds respond to desiccation (Leprince, 2004; Ballesteros *et al.,* 2020). External factors such as the rate of drying according to water potential gradients and temperatures applied to seeds (Leprince, 2004), as well as post-harvest handling (Probert *et al.*, 2007), also affect desiccation responses. Controlling all these factors is key to seed conservation, including for wild species (Hay and Probert, 2013).

**Figure 1:**
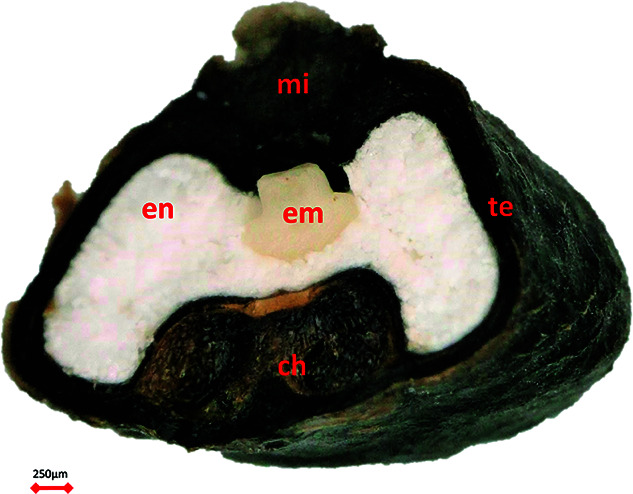
*Musa acuminata* seed. mi, micropyle cap; en, endosperm; em, embryo; te, testa; ch, chalazal mass.

Wild banana species produce fruit that contains hard dark seeds (Fig. 1). These seeds have two chambers: the larger chamber contains the capitate embryo and powdery endosperm and the other chamber contains a chalazal mass at the point of the former ovule where the integuments were united to the nucellus. A micropyle, filled by a micropyle cap, is located above the embryo. Banana seeds are variously considered orthodox (Chin, 1996; Singh *et al*., 2021) or intermediate in class (Chin and Krishnapillay, 1989; Darjo and Bakry, 1990; Abdelnouresquivel *et al*., 1992; Bohra *et al*., 2020). Either way, viability of several *Musa* seed genebank collections is low (Kallow *et al.,* 2020; Kallow *et al.,* 2021a; Kallow *et al.,* 2021b) and seed conservation is therefore constrained (Laliberté, 2016; Panis *et al*., 2020).

While seed conservation of wild species, including CWRs, holds many challenges compared to crops (Hay and Probert, 2013), a key one, especially for banana, is access to seeds. This is because it is challenging to find mature seeds in wild populations and difficult to arrange transfer of genetic material between countries (Panis *et al.,* 2020). The objective of this work was to assess if drying can be used in *ex situ* seed conservation of banana CWRs. Here we describe four experiments performed in three countries (Belgium, Viet Nam and the Philippines). With these we examined responses to drying in seeds of the two primary CWRs of banana. We aim to address the following questions: (i) To what extent are seeds desiccation tolerant? How does (ii) maturity, (iii) post-harvest ripening and (iv) drying rate affect desiccation tolerance? (v) How does dehydration affect embryo shrinkage of different maturities?

## Materials and methods

### Plant material

We used open pollinated seeds of eight bunches (infructescences) in four experiments (Table 1; Fig. S1). Seeds were of two species of *Musa acuminata* Colla (including three subspecies) and *Musa balbisiana* Colla. These species most closely relate to banana crops (De Langhe *et al*., 2009).

**Table 1:** Origin of plant material used (see Fig. S1 for images)

Bunch	Taxa	Accession	Location	Institution	Date	Seed maturity	Fruit ripeness	Experiment
*1*	*M. acuminata* subsp. *siamea*	PT-BA-00262	Roujol, Guadeloupe	CIRAD	07/2020	Mature	Ripe	1
*2*	*M. acuminata* subsp. *banksii*	PT-BA-00412	Roujol, Guadeloupe	CIRAD	07/2020	Mature	Ripe	1
*3*	*M. acuminata* subsp. *microcarpa*	PT-BA-00287	Roujol, Guadeloupe	CIRAD	08/2020	Mature	Ripe	1
*4*	*M. balbisiana*	GB61996	Los Baños, Philippines	UPLB	10/2019	Mature	Ripe	2
*5*	*M. balbisiana*	GB61996	Los Baños, Philippines	UPLB	10/2019	Less mature	Unripe	2
*6*	*M. balbisiana*	VTN785	Êa Trul, Viet Nam	PRC	01/2020	Mature	Mixed	3
*7*	*M. balbisiana*	PT-BA-00019	Roujol, Guadeloupe	CIRAD	10/2020	Mature	Ripe	4
*8*	*M. balbisiana*	PT-BA-00019	Roujol, Guadeloupe	CIRAD	10/2020	Less mature	Unripe	4

Bunches from Guadeloupe (bunches 1–3 and 7–8) were collected from living accessions of the field collection at the Centre de Coopération Internationale en Recherche Agronomique pour le dévelopment (CIRAD), Roujol, Guadeloupe. Bunches were air shipped to Leuven, Belgium, where certain experiments were performed. Transportation took <7 days and temperatures were >0°C and <25°C during shipment; humidity was not controlled during shipment. Bunches 1–3 were collected as mature green fruit, and after arrival in Leuven (Belgium) fruit had ripened and were yellow to brown and black and were soft in texture; seeds were hard, with powdery white endosperms and capitate endosperms. Bunches 7–8 were deliberately collected at two levels of maturity from the same accession in the field genebank. Bunch 7 was very mature and ripe with brown-black soft fruit on arrival. Seeds were hard with powdery endosperms and capitate embryos. Bunch 8 was less mature and ripe on arrival with soft yellow fruit. Again, seeds were hard, but with less hard seed coats on biting and endosperms were white and powdery with capitate embryos. An additional third bunch was also collected from the same accession at an even less mature stage of development, fruit were light green and hard, embryos were not capitate and endosperms were milky, so seeds of this bunch were excluded from the experiments. Seeds were extracted by opening fruit by hand and washing the seed and surrounding pulp in tap water to remove pulp and any empty seeds. Resulting seeds were then spread out on newspaper for 24 hours for evaporation of surface water (~20°C, 60% RH). Seeds from the various fruit (berries) and hands (groups of fruit from the former clusters of flowers subtended by one bract) were pooled.

Bunches from the Philippines (bunches 4 and 5) were collected from the field collection of the Institute of Plant Breeding, University of the Philippines Los Baños (UPLB), where experiments were also carried out. These bunches were of the same accession growing from neighbouring mats at two levels of maturity. Both bunches contained firm dark seeds, such as would be harvested during field collection missions. The more mature bunch had fruit that were orange to brown, it also had some hard green fruit that contained no seeds. Approximately half of the fruit containing seeds were already open when the bunch was collected. Pulp of fruit containing seeds was soft and silky in closed fruit or brown and grainy in open fruit. Seeds were black and hard, perceptibly harder than seeds from the less mature fruits when bitten. Embryos were capitate in shape and endosperms were dry and powdery, even after washing. Fruit from the less mature bunch were green and these contained many seeds. Fruit texture was hard. Seeds were hard and black and could not be crushed by fingernails but could more easily be crushed when bitten. Seeds also had capitate shaped embryos. Endosperms were dry and white, but not powdery and easily absorbed moisture during washing so that the endosperm became flooded with water. Seeds were extracted and cleaned as described above.

Bunch 6 was collected from a home garden in Êa Trul commune (Buôn Đôn district, Đ$\textrm{a}\!\!^{\acute\breve}\;$k L$\textrm{a}\!\!^{\acute\breve}\;$k province, Viet Nam) during a collecting mission. Seeds were extracted and cleaned as described; the drying part of the experiment was carried out during the field mission in Đ$\textrm{a}\!\!^{\acute\breve}\;$k L$\textrm{a}\!\!^{\acute\breve}\;$k province and seeds were sown at Plant Resources Center, Ha Noi, Viet Nam. The bunch was discovered after having been previously removed from the plant for flower harvesting for food, so we do not know how long had passed after removal from the plant. Fruit on the bunch were at two levels of ripeness: the lateral part of the bunch in contact with the compost it was lying on was ripe and the lateral part of the bunch exposed directly to the air was still unripe. Ripe fruit differed from unripe in that they were yellow-brown in colour compared to light green and soft compared to hard. Seeds from all fruit were hard when pinched, and endosperms were powdery. Seeds had capitate embryos in all seeds examined. Fruit from both ripe and unripe fruit contained many seeds per berry. Extraction and processing were carried out in the field, and therefore under inherent constraints.

### Experiments

#### Experiment 1: the effect of relative humidity on moisture content and viability

Seed survival, MC and equilibrium relative humidity (eRH) of three *M. acuminata* subspecies (bunches 1–3; Table 1) were assessed before and after equilibrating seeds to seven levels of RH. MC and eRH were also assessed directly after extraction from fruit pulp, without any washing or surface drying. eRH was measured using a hygrometer (HygroPalm AW1, Rotronic, Bassersdorf, Switzerland). A sample cup (height, 40 mm) was filled with seeds from each bunch/treatment combination, and the RH was measured after equilibrating for 20 minutes. MC was measured by weighing seeds before and after dehydration at 70°C for 3 days in an oven (non-standard temperatures and extended drying time was used because of oven availability following experimental verification that dry weight was achieved for *Musa* seeds) and then calculating MC percentage (i.e. on fresh weight basis). Three batches of 10 seeds were used for each bunch/treatment combination, and mean MC was calculated. Five of the seven levels of RH were achieved by equilibrating seeds in sealed humidity controlled chambers, regulated with non-saturated lithium chloride (LiCl) solutions (11, 15, 20, 40, 65% RH; Table S1) (Hay *et al*., 2008; Gold and Hay, 2014). The final RH (100%) was achieved by only adding deionized water to the chamber. Chambers were 325 ml in capacity, using 65 ml of solution, sealed with cling film and equilibrated at 20°C for 3 days prior to use. RH in chambers was tested using a hygrometer before suspending seeds above the solution on wire shelves avoiding contact with the solution. The seventh RH level was achieved using silica gel in a desiccator. The RH of the desiccator chamber was measured with a hygrometer in the chamber with seeds. Seeds were equilibrated in chambers for 14 days, at 20°C and subsequently removed for assessment of MC and viability.

Seed viability of each bunch/treatment combination was measured using three methods. One, the tetrazolium chloride (TTC) test, using a 0.5% 2,3,5-triphenyl-2H-tetrazolium chloride solution, and 24 seeds from each bunch/treatment. After removal from humidity-controlled chambers, seeds were imbibed for 48 hours on 1% agar in a Petri dish at 20°C. Embryos were then removed from seeds using forceps and scalpel and submerged in TTC solution divided between two 1.5 ml Eppendorf tubes per bunch/treatment. These were incubated for 48 hours in the dark at 27°C. Embryo staining was assessed under a binocular microscope. Tetrazolium chloride is a colourless compound that stains living tissues red when it is enzymatically reduced to triphenyl formazan. Embryo colouration was categorized into four groups: full red, red coloration at the tip (embryonic axis), pink or no coloration.

The second viability method was based on ER. All ER procedures were completed in sterile conditions, in a laminar flow hood using the method described by Kallow *et al.* (2020). Embryo reactions were categorized 28 days after initiation, as follows: germinated (showing shoot growth), callused, blackened, contaminated with fungi or bacteria or showing no growth.

The third viability test method was whole seed germination test. For this we placed 24 seeds per bunch/treatment combination in a Petri dish with 50 g of sterilized sand moistened with 7 ml of deionized water. Petri dishes were then placed in an incubator at alternating temperatures of 20°C for 18 hours in the dark and 35°C for 6 hours in the light continuously cycled for 6 weeks (based on Stotzky *et al*., 1961 and Kallow *et al.,* 2021a). Germination was scored every 7 days; any germinated seeds showing radicle emergence were recorded and removed.

A seed moisture desorption isotherm was plotted for seed MC and eRH using non-parametric local polynomial regression for curve fitting (Loess). Multinomial logistic regression (MLR) with counts according to ER and TTC outcomes were modelled separately with treatment RH and subspecies/accession as factors, tested in a minimum adequate model (MAM) using ANOVA and *X^2^* test. Post hoc estimated marginal means were calculated for each bunch/treatment combination and contrast analysis carried out using the Dunnett test against the control treatment (superficial surface-dried seeds). All statistical analyses were carried out in R (R Core Team, 2020).

#### Experiment 2: the effect of maturity on viability and drying

Initial seed viability of bunches 4 and 5 at two levels of maturity was tested by ER after surface drying for 24 hours in ambient laboratory conditions and for a further four consecutive days of desiccation. Seeds were desiccated by placing them in a desiccator (inner diameter, 250 mm) with dried silica gel (~1500 cm^3^). A sample from each bunch remained in ambient conditions in the laboratory and was tested alongside desiccated seeds on the final day. Forty seeds per bunch were tested at each time point. At each time point and immediately on extraction (without washing), MC of seeds was assessed as described above, except in these instances dry weight was achieved at 103°C for 17 hours. Additionally, the MC of fruit pulp of the two bunches was tested in three replicates of ~1 cm^3^ of pulp without washing.

To assess the capacity of fresh whole seeds to germinate, surface-dried seeds, maintained at ambient conditions for 5 days, were sown in two plastic trays (100 × 40 × 10 cm). Trays were divided in half, with seeds from different bunches sown at either side of the tray. Seeds were sown in soil (clay loam) and were placed in a light screen house at UPLB. Seeds were covered with 5 mm of substrate; trays were placed on a concrete path and watered each day. Two replicates of two hundred seeds were sown from each bunch. Trays were checked each day and germinated seedlings were recorded and removed. The germination test was concluded after 55 days.

Mean MC of freshly extracted mature and less mature seeds (without washing) was compared using a t test, as were mature and less mature seeds after 4 days in the desiccator. ER and final seed germination was converted to final percentage germination for descriptive purposes. ER responses to drying were assessed using a binomial generalized linear model (GLM) with MC as explanatory variable.

#### Experiment 3: the effect of fruit ripeness and drying rate on seed germination

Seeds from ripe and unripe fruit of the same bunch were kept separately. Seeds were dried in paper sachets in three conditions: ‘air dry’ (paper bag no silica gel), ‘slow dry’ (sealed in zip-loc bags containing one sachet of silica gel; total volume, 20 cm^3^; GeeJayChemicals, UK, self-indicating orange to colourless, 1–3 mm), ‘fast dry’ (sealed in zip-loc bags containing six sachets filled with silica gel; total volume, 6 × 20 cm^3^). Seed eRH was monitored with a hygrometer (HygroPalm AW1, Rotronic, Bassersdorf, Switzerland). Seeds with silica gel were dried to 35–40% eRH, as measured by the hygrometer, then the silica gel was removed (~39 hours for fast drying, 112 hours for slow drying; Fig. S2). Seeds were maintained in paper bags in ambient conditions until sown 2 weeks later, therefore only the drying rate to 35–40% eRH was controlled for slow and fast-dried seeds. Seeds dried without silica gel reached a minimum of 65% eRH (from ripe fruit) or 68% eRH (for unripe fruit). Germination testing was then carried out in the soil, at the Plant Resources Center, Ha Noi, Viet Nam. Four replicates of 40 seeds per ripeness/treatment combination were directly sown in the soil and covered by 1 cm of soil. Seeds were sown in four replicate 50 cm^2^ grids per sample in the same sowing area. A control set of seeds, from mid-way between the green and yellow-brown fruit of the same bunch, were extracted. These had been transported back to the centre while still attached to the bunch pedicle inside a large plastic bag and were also sown. The sowing area was monitored every 2 days and germinated seedlings were recorded per replicate and removed.

Seed germination response was tested using a binomial GLM, with ripeness and drying as factors. The MAM was found by removing interactions and factors and comparing models using ANOVA and the *X^2^* test. Post hoc contrast analysis was performed on the MAM by calculating estimated marginal means and standard errors and using the Dunnet test with treatments against control.

#### Experiment 4: the effect of drying and imbibition on seed morphology

Eight seeds from each of two bunches (bunches 7–8), of the same *M. balbisiana* accession, were randomly selected. Seeds were then scanned for visualization and measurement of the embryo volume after surface drying (detailed X-ray computer tomography (CT) scanning and image analysis methods are in the Supplementary Methods), and were then divided between two RH chambers, at 15% RH and 100% RH was controlled as described above. Seeds were equilibrated in chambers for at least 14 days. Then, two seeds per bunch/chamber were re-scanned, and two seeds per bunch/chamber were re-imbibed for 7 days at 20°C on agar and re-scanned. Additionally, ER was conducted, as described above, on 24 seeds in each of these same conditions (surface dried only, 15% eRH, 100% eRH, 15% eRH and re-imbibed, 100% eRH and re-imbibed).

Repeated measures linear mixed effects models were used to compare changes in volume during RH equilibration and re-imbibition. Again, post hoc estimated marginal means and contrast analysis using the Dunnett test was used against the control. Finally, all five ER responses were assessed using MLR, followed by post hoc estimated marginal mean calculation and Dunnet test against the fresh seeds.

## Results

### Experiment 1

**Figure 2:**
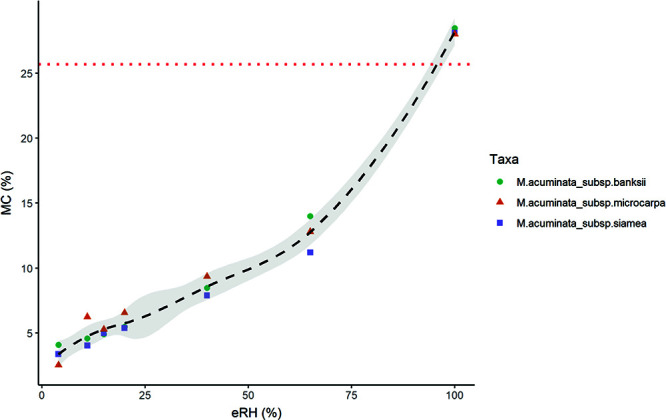
Desorption isotherm for seeds of three *M. acuminata* subspecies (bunches 1–3). Seeds were equilibrated in RH-controlled chambers using non-saturated lithium chloride, apart from 4% RH in a desiccator with silica gel and 100% RH with water. The black dashed line is fitted with non-parametric local polynomial regression; the grey shaded area is 0.95 confidence interval; the red dotted horizontal line is mean seed MC directly at extraction without washing. MC calculated on fresh weight basis. No statistically significant differences were found between bunches/subspecies so they are combined in the trend line here.

MC of seeds of three subspecies of *M. acuminata* ranged from a maximum of 28% at 100% eRH, to a minimum of 2.5% at 4% eRH in the desiccator. Freshly extracted seeds, without washing, were close to the maximum, 26 ± 3% MC (mean, standard deviation, used hereon). There was very little difference between subspecies (thus excluded from the MAM). A desorption isotherm was fitted to the MC and eRH values (Fig. 2). Three phases of water desorption (Vertucci & Roos, 1990; McDonald, 2007) were apparent: a convex region at RHs below 20%, a linear region at RHs between 20 to 65% and a concave region at RHs above 65%.

**Figure 3:**
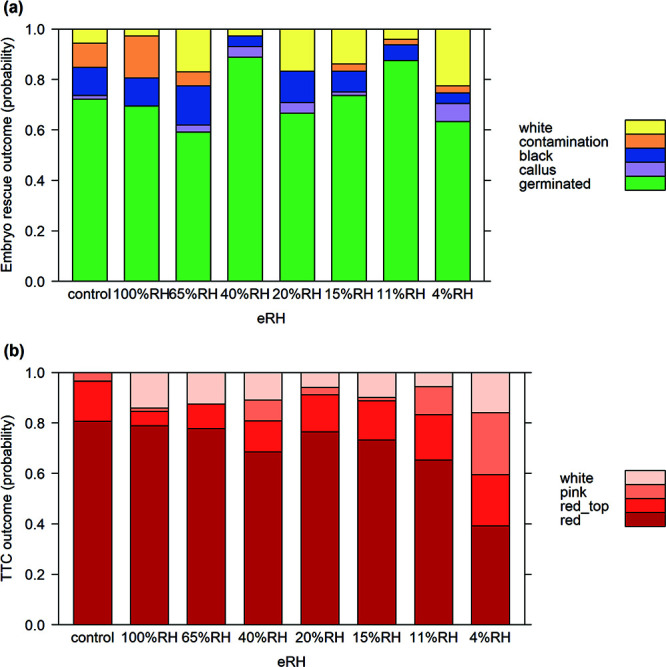
(**a**) ER outcomes and (**b**) tetrazolium chloride outcomes, modelled from MLR based on seeds (bunches 1–3) equilibrated to different RHs.

ER outcomes showed no significant contrasts against the control (superficially surface-dried seeds) according to MLR and Dunnett tests for all apart from an increase in embryos showing no reaction (white) at 4% eRH (*t* = 3.007, df = 32, *P* = 0.029) and a lack of contamination in 40 and 20% eRH (*t* = −2.785, df = 32, *P* = 0.0496; Fig. 3a). The TTC tests also showed significant effects when seeds were at 4% eRH (Fig. 3b): red-stained embryos reduced (*t* = −5.749, df = 24, *P* < 0.001) and pink-stained embryos increased (*t* = 4.834, df = 24, *P* = 0.0049). Across all treatments, apart from 20% and 11% eRH, there were more embryos showing no staining compared to the control that had none showing no staining. For both ER and TTC models subspecies/accession were excluded from the MAM. None of the whole seeds in either the control groups or any of those equilibrated to different eRHs germinated in the incubator at 35/20°C.

### Experiment 2

**Figure 4:**
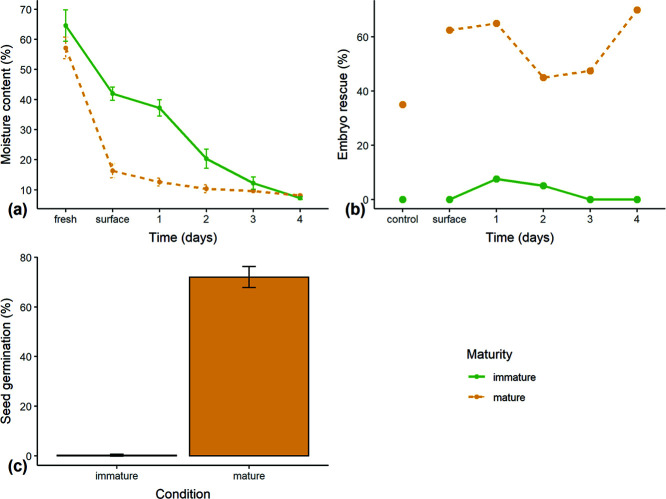
Results from seeds of two bunches of *M. balbisiana* at different levels of maturity (bunches 4–5). (**a**) MC (fresh weight basis) during desiccation in a desiccator with silica gel; ‘fresh’ is without washing, ‘surface’ is after 24 hours surface drying in ambient laboratory conditions after washing, otherwise days are time in a desiccator (*n* = 3 × 10). (**b**) ER results as percentage of sample forming shoots within 28 days of initiation samples removed from a desiccator each 24 hours; ‘control’ seeds were left in ambient conditions for 4 days (*n* = 40). (**c**) final germination results 55 days after sowing in soil. Whole seeds were sown and seedlings were recorded and removed daily; seeds were covered with 5 mm of soil and exposed to sun in a screen house; seeds were 5 days after extraction maintained at ambient conditions without desiccation (*n* = 200).

There were no significant differences in MC of freshly extracted mature and less mature seeds without washing or superficial drying (mature seeds: 57 ± 4% MC, less mature seeds: 65 ± 5% MC, *t* = −2.029, df = 3.562, *P* = 0.121; Fig. 4a). However, mature seeds dried faster, especially during surface drying and the first 24 hours desiccation (mature seeds: 16.3 ± 2.4% MC, less mature seeds: 42.0 ± 2.2% MC, *t* = −13.863, df = 3.974, *P* < 0.001). After 4 days desiccation mature seeds had a small but significant higher MC compared to less mature seeds (8.1 ± 0.1% and 7.3 ± 0.4%, respectively; *t* = −13.863, df = 3.974, *P* < 0.001). Overall seeds from the less mature bunch therefore lost a small but significantly greater amount of moisture during desiccation. The pulp of the mature bunch was 74 ± 0.5% MC and the less mature pulp was 83 ± 1.1% MC.

Mature seeds were desiccation tolerant to the minimum level of desiccation achieved in the timeframe (8% MC; Fig. 4a, b). Initial germination of mature seed that had been only surface dried was 63%, after 5 days desiccation germination was 70%. Control seeds maintained in ambient conditions lost viability to 35% after 5 days. There were no significant germination differences according to MC for mature seeds (*z* = 1.766, *P* = 0.077). Seeds of the less mature bunch showed 0% germination after surface drying, the maximum germination achieved was 8% after 1 day in the desiccator, but no germination beyond 2 days in the desiccator. In the soil germination tests, mature seeds had a high final germination (72 ± 4%), compared to the very low germination of less mature seeds (0.25 ± 0.35%, Fig. 4c).

### Experiment 3

**Figure 5:**
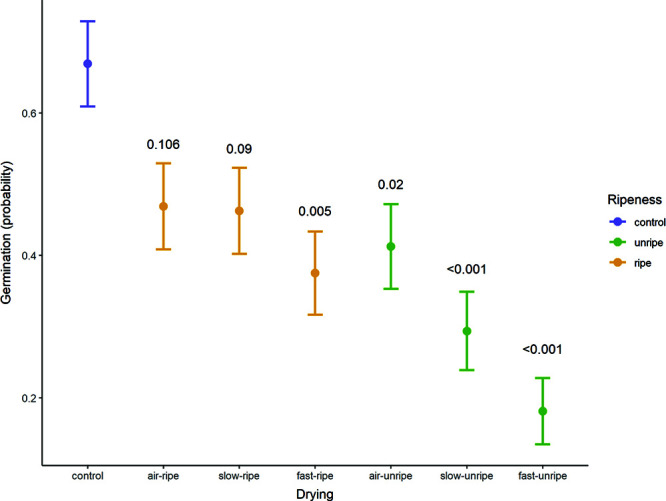
Estimated marginal means of germination based on GLM with quasibinomial error structure; estimates back-transformed to probability scale. *P*-values (shown above error bars) are contrasts against control seeds that had not been dried. *M. balbisiana* seeds used (bunch 6), fresh and after three levels of drying and two levels of ripeness. Control seeds were extracted 2 weeks later on return to the laboratory. Seeds were sown in the soil at Plant Resources Center, Ha Noi, Viet Nam, and monitored for 78 days.

Ripeness and drying rate (amount of silica gel during drying to 35–40% RH) had a significant effect on final germination in the GLM (Fig. 5). Contrast analysis showed that seeds from unripe fruit in all drying conditions, and ripe fruit fast dried only, had significantly lower germination than control seeds that had not been dried (air dry: *z* = −2.898, *P* = 0.020; slow dry: *z* = −4.177, *P* < 0.001; fast dry: *z* = −5.344, *P* < 0.001). Air-dried and slow-dried seeds from ripe fruits were not significantly different than fresh control seeds (air dry: *z* = −2.285, *P* = 0.101; slow dry: *z* = −2.354, *P* = 0.09). Within ripeness category, drying rate always had an effect in the order: air, slow, fast. Quasibinomial error structure was used because of overdispersion of binomial models.

### Experiment 4

**Figure 6:**
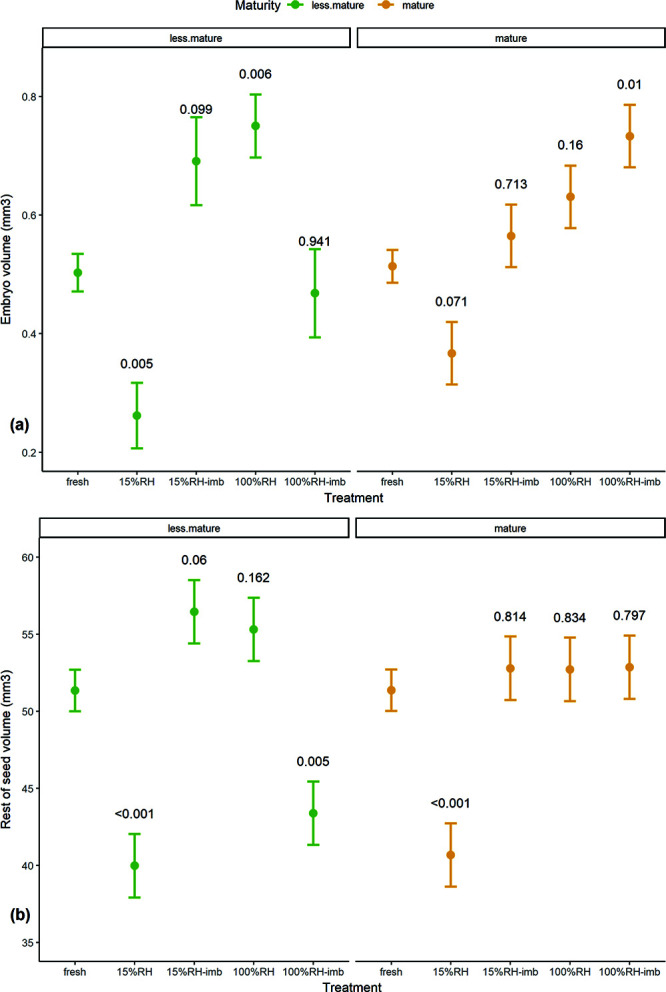
Estimated marginal means (and standard errors) of embryo (**a**) and seed volume excluding embryo and air space (**b**), based on repeated measures linear mixed model. *P-*values shown from Dunnett’s test against fresh seeds (‘fresh’), for seeds equilibrated at 15% RH and 100% RH for at least 14 days without imbibition and with imbibition on agar for 7 days (‘imb’) (bunches 7–8).

**Figure 7:**
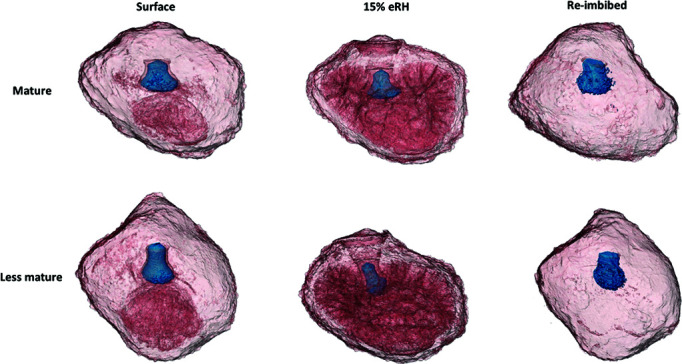
A 3D visualization of *M. balbisiana* seeds (bunches 7–8) from CT scans. Displayed seeds were either surface dried only and left in ambient conditions or equilibrated to 15% RH for 14 days and then either scanned immediately after removal from 15% RH conditions or re-imbibed for 7 days on agar at 20°C. Embryos are coloured blue; red shows empty surfaces within the seed where moisture has been desiccated.

The volumes of the embryo and the rest of the seed (excluding air space and embryo) were visualized by CT scanning seeds. We then calculated volumes before and after equilibrating at 15% RH and 100% RH, and then again after re-imbibing seeds. We did this for seeds of mature and less mature bunches collected from the same *M. balbisiana* accession. We found that embryos from less mature seeds significantly reduce volume when dried (*t* = −4.000, *P* = 0.005; Figs 6a and 7) and increased volume when equilibrated at 100% RH (*t* = 4.741, *P* = 0.006). By contrast embryos of mature seeds do not significantly change in volume when dried (*t* = −2.806, *P* = 0.071). Embryos of mature seeds also significantly increase in volume after equilibrating at 100% RH and then on agar for 7 days (*t* = 4.203, *P* = 0.01), but less mature embryos do not (*t* = −0.470, *P* = 0.941). Notably, when the embryo volume is decreased on desiccation, the airspace around the embryonic axis in the micropyle is evident, noticeably for less mature seeds (Fig. 7).

Drying caused the seed (not counting the embryo) to reduce in volume for both mature and less mature seeds (less mature: *t* = −6.296, *P* < 0.001; mature: *t* = −5.915, *P* < 0.001; Fig. 6b). In particular, the endosperm and chalaza mass lost volume that was replaced by air space (Fig. 7). Notably, this effect was evident in the chalaza mass for surface-dried seeds compared to re-imbibed seeds, meaning the chalaza mass is the first part of the seed to lose moisture, and also to take up moisture on re-imbibition.

**Figure 8:**
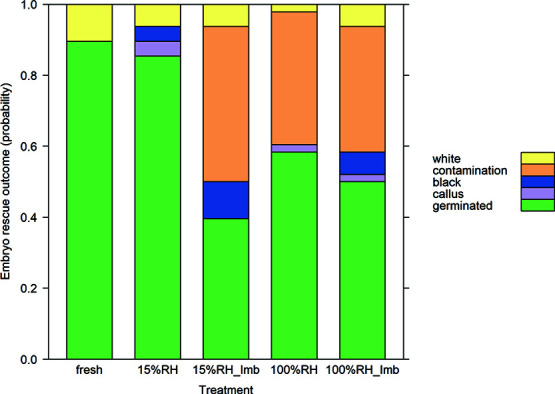
Plot of MLR model of ER results of both mature and less mature *M. balbisiana* seeds (bunches 7–8) used in experiment 4.

There was no difference in ER results according to the maturity of the bunches used in the CT scan; the minimum adequate MLR model contained only treatment and not maturity. Post hoc Dunett tests showed germination decreased when seeds were equilibrated at 100% eRH (for 14 days) and also if they were re-imbibed for 7 days after equilibrating to 15% RH (Fig. 8). The probability that an embryo would germinate is replaced by contamination in these treatments. There was no significant change in embryo outcome between fresh seeds and those at 15% eRH (without re-imbibition), meaning viability is lost due to contamination and not desiccation sensitivity in the embryo for mature and less mature seeds in this experiment.

## Discussion

Seed responses to desiccation are essential to seed genebanking. In the present study we examined *Musa* seed responses to drying, ultimately to inform best practice for conservation practitioners.

### Moisture content

The desorption isotherm of banana presented here (Fig. 2) is comparable to other starchy seeds (McDonald, 2007). Seed physical and physiological changes correlate with seed oil content during drying and re-imbibition because water binding depends on seed chemical properties. All seeds typically show three water-binding regions and these are remarkably similar according eRH, but the MC of these depends on seed oil content (Vertucci and Roos, 1990). Region 1 is found below 15–20% eRH, where only strong binding water remains in the seed and the seed is in quiescence; region 2 is situated between 20% and 90% eRH where there is also weak binding water and seed ageing increases with RH and temperature; and region 3 is >90% eRH where respiration and repair can take place (Sun, 2002; McDonald, 2007). The FAO Genebank Standard guidance is for seeds to be dried to 10–25% eRH, depending on species (FAO, 2014), i.e. around the cusp of regions 1 and 2. Our isotherm for *M. acuminata* (Fig. 2) shows transition from region 1 to 2 at 15–20% eRH (at 20°C) or 5–6% MC. We anticipate that isotherms are similar across the Musaceae as they all exhibit powdery starchy endosperms and gelatinous chalazal masses (Fig. 1) (Benedict *et al.,* 2016). Further moisture profile assessments of the embryo would also be beneficial, as the isotherm presented refers to the whole seed.

The chalaza was the first part of a seed to dry in ambient conditions (Fig. 7). The chalaza therefore acts as a buffer, regulating moisture in the rest of the seed including the embryo, thereby limiting the effect of sudden drying or slowing initial drying after dispersal from the high moisture of the fruit (Kiew, 1987). Drying of the chalazal mass has also been proposed to facilitate secondary dormancy in banana seeds (Chin, 1996); however, our results show seeds readily re-imbibe moisture. Additionally, the chalazal quickly absorbs moisture during imbibition, this increases pressure and contact between the endosperm and embryo, and therefore allows uptake of sugars by the embryo.

### Desiccation tolerance

Our data show that both *M. acuminata* and *M. balbisiana* seeds can be dried to 15% eRH (5% MC) and even down to 4% eRH (2.5% MC) without any loss of ER viability (Figs 2 and 8). This finding is broadly in keeping with recent results for *M. balbisiana* (Singh *et al.,* 2021), where seeds were desiccation tolerant to ~10% MC, dried in a laminar flow or room temperature, but not with silica gel (to 7% MC). Another study found *M. acuminata* embryos were desiccation tolerant to 15% MC, but lost around half of their viability when dried to 6% MC (Abdelnouresquivel *et al.,* 1992). Loss of viability in storage at 15% eRH or when dried in a desiccator with silica gel to 2.4% MC has also been reported for six *Musa* species, although *M. balbisiana* was found to have greater desiccation tolerance than others in that instance (Kallow *et al.,* 2020; Kallow *et al.,* 2021a). Desiccation tolerance in *Musa* is therefore possible but not consistent and relies on other factors discussed below.

### Maturity

Maturity had a profound effect on viability (Fig. 4), less mature seeds showed negligible viability, as evident in both ER and germination tests. These seeds were seemingly well developed, with capitate embryos, white and powdery endosperms and hard dark seed coats. This is particularly notable as germination capacity develops after embryogenesis and seed filling, and prior to late maturation, important for desiccation tolerance (Leprince *et al.,* 2017). Visually observed well-developed seeds (in experiment 2) had not even reached the stage where germination was possible, let alone survive desiccation. These results contrast with those of experiment 4, where less mature seeds (ripened during shipment) had high germination and maintained viability even after desiccation to 15% eRH.

In comparable studies, less mature wild collected *M. acuminata* subsp. *banksii* seeds had minimal ER germination that reduced further on drying (Kallow *et al.,* 2020). Seeds of *M. balbisiana* were required to be fully mature at 125 days after flowering (DAF) for any whole seed germination and optimal ER, although some ER germination was achieved at 100 DAF (Singh *et al.,* 2021). Seeds of *M. acuminata* needed 115 DAF for maximum germination (Darjo and Bakry, 1990) and 99 DAF meant embryos produced shoots rather than callus (Uma *et al*., 2011). Additionally, Simmonds (1952) found that *M. balbisiana* seeds germinated to >80% from 4 weeks before and 2 weeks after ‘maturity’ (maturity was undefined, fruit were also ripened). Simmonds also found an interaction between maturity and desiccation tolerance, being more apparent for *M. acuminata* than for *M. balbisiana,* the latter again seems to be less sensitive to drying.

Desiccation sensitivity, related to incomplete structural maturation, is certainly indicated in our results, as embryos of less mature seeds shrank significantly during drying, in comparison to mature embryos that did not (Figs 6 and 7). During the latter stages of maturation of orthodox seeds, seeds dry after abscission. During this stage embryo cell vacuoles decrease in size and insoluble reserves deposit in the cytoplasm, such as longer-chain non-reducing sugars, late embryogenesis heat shock proteins and antioxidants are accumulated (Berjak and Pammenter, 2008). These structural changes in orthodox seeds prevent massive cell shrinkage and mechanical strain during desiccation and therefore may play a crucial role in protecting cells during desiccation (Perez *et al.,* 2012; Walters, 2015; Ballesteros *et al.,* 2020). This was observed in the difference of volume change in mature and less mature embryos.

The maturation process has most thoroughly been described for dry-dispersed orthodox seeds (Ellis, 2019). However, seeds of fleshly fruit show similar processes of maturation drying after mass maturity (Demir and Ellis, 1992a; Demir and Ellis, 1992b). Freshly extracted seeds of mature and less mature seeds were not significantly different in MC (Fig. 4); however, after removal of surface moisture with 24 hours of ambient drying they were remarkably different. This therefore suggests that maturation drying is required, not only for germination but also for desiccation tolerance.

The challenge for seed conservation of *Musa* is how to reliably identify desiccation-tolerant seeds in the forest, given maturation is environmentally regulated (Daws *et al.,* 2006; Ellis, 2019) and DAF cannot be known in these settings and are season dependent. Dry mass accumulation and change in fruit colour are used by seed collectors to identify seed maturity in fleshy fruits like bananas (Hay and Smith, 2004). Additionally, a hard seed coat with fused integuments, a powdery endosperm and capitate embryo have been identified here and elsewhere as indicators (Kallow *et al.,* 2020; Singh *et al.,* 2021). However, more clear morphological guidance is needed for seed collectors.

### Ripeness

Post-harvest ripening increased desiccation tolerance (Fig. 5). Seeds from ripened fruits showed higher germination frequencies and were less susceptible to harsher, faster drying rates than seeds in unripe fruits. This was also evident in bunches 1–3, whose desiccation tolerant seeds had ripened during transit. In each of these instances ripening occurred on the peduncle after harvest, whereas in another study seeds showing low desiccation tolerance were shipped as loose fruits or hands (Kallow *et al.,* 2020).

Simmonds (1952) found that seeds of *M. acuminata* and *M. balbisiana* were less susceptible to drying if fruit were ripened (presumably after harvesting) and ripening had a large effect if the bunch was less mature compared to mature. Although, in another experiment, seeds did not show desiccation sensitivity and ripening to ‘black-ripe’ and ‘rotted’ had a negative effect on germination before and after drying. Simmonds (1959) also found that drying ripe and over-ripe whole mature fruits in the oven for 4 days at 45°C had a surprisingly positive effect on viability, but for unripe fruit this was not the case.

Post-harvest maturation of seeds collected before mass maturity has been observed for several species and is typically optimal under conditions approximate to the natural environment (Hay and Probert, 1995; Lima *et al*., 2005; Probert *et al.,* 2007; Whitehouse *et al.*, 2017). Presumably, the ripeness of fruit in experiment 3 was encouraged through the microclimate and chemical exchange with the decomposing matter at the lower side of the bunch; and fruit in experiments 1 and 4 ripened during transit. Banana ripeness is primarily controlled by ethylene and ABA (Thompson *et al.*, 2019) and involves conversion of starch to sugars in fruit in order to attract dispersers (Marriott *et al.*, 1981). As experiment 3 was carried out during a field mission, with a bunch collected in an uncontrolled manner, the environment in which the ripe part of the bunch developed was not known. Being able to extend the small window between maturation and dispersal by post-harvest maturation is very useful for wild seed collecting, and therefore ripe for further research.

### Rate of drying

Seeds had better viability when dried slowly compared to faster drying (to 35–40% eRH, ~8.6% MC), particularly when fruit were unripe (Fig. 5). This is in keeping with Singh *et al.* (2021) who found that *M. balbisiana* seeds had high viability when dried either at room temperature (minimum, 9.8% MC) or in the laminar flow (minimum, 19.5% MC) or a combination of these (minimum, 10% MC), but not in a desiccator with silica gel (minimum, 4.1% MC). However, seeds maintained high viability when dried in silica gel if they were pre-dried with a combination of laminar flow and room temperature (10% MC). Then they maintained high viability after further drying with silica gel (89% ER, 5.5% MC).

Slow drying seeds is beneficial for some (Greenwood and Stark, 2014; Stark *et al.,* 2016), but not all species (Wesley-Smith *et al.*, 2001). In our results, it was seeds of unripe fruits that were most sensitive to drying rate, and this is in keeping with other studies for immature seeds prior to post-abscission maturation (Hay and Probert, 1995; Hong and Ellis, 1997; Lima *et al.*, 2000; Lima *et al.,* 2005; Whitehouse *et al.*, 2015).

### Implications for conservation

Conserving CWRs is a global priority (CBD, 2012; FAO, 2021). It protects genetic resources for crop adaptation and breeding and therefore aids food security. For bananas this impacts the many millions of people who rely on this crop for food and income in the face of environment change. *Ex situ* seed conservation is the most efficient form of *ex situ* conservation for bananas and allows easy access of material for use (Panis *et al.,* 2020). Our results show that to maximize seed viability, and therefore the value of *ex situ* collections, care is needed at the collection stage, ensuring seeds are collected at full maturity and fruit is allowed to ripen. We have demonstrated some of the morphological characteristics that identify mature fruit and seeds that signal seed maturity. These are in keeping with general advice for seed collectors that seed should be collected at the point of natural dispersal (Hay and Smith, 2004; Hay and Probert, 2011). For bananas this is often a challenge because of frugivory; however, it is evidently a false economy to collect and store material that does not survive in storage, so solutions need to be found in protecting seeds from frugivory to allow for collection of mature seeds from wild populations.

Additionally, the present study investigated the effect of drying on seed storage; however, storage temperature is also an important consideration for survival and longevity. Initial results elsewhere show that *Musa* seeds survive for at least 5 years storage at a range of temperatures (25°C, 5°C, −20°C and − 196°C) if they are adequately dried to below 10% MC (Panis *et al.,* 2020; Singh *et al.,* 2021).

To extend and consolidate our findings further investigation, with a wider range of species, would be beneficial; very few *Musa* species have been investigated for storage behaviour. Access to suitable material was a major constrain to the present study, meaning we had to work in several locations and laboratories. This limited consistency in the experiment methods used, such as seed collection, cleaning and desiccation, and demonstrates some of the challenges with regard to conservation of plant genetic resource (Engels and Thormann, 2020; Pearce *et al.,* 2020).

## Conclusions

Conservation of plant genetic resources, including seed genebanking, is important for global food security to meet the challenges of the future. Our results show responses to drying in banana wild relatives. We conclude that (i) *Musa* seeds are desiccation tolerant; (ii) seeds must be fully mature to germinate, even for ER; (iii) ripening reduces desiccation sensitivity; (iv) fast drying decreases survival, this effect is reduced if seeds are ripened; (v) less mature embryos undergo greater shrinkage, indicative of desiccation sensitivity. Based on our results, we anticipate seed conservation will become an *ex situ* conservation method for banana CWR genetic resources.

## Funding

This work was supported, in whole or in part, by the Bill & Melinda Gates Foundation ‘BBTV mitigation: Community management in Nigeria, and screening wild banana progenitors for resistance’ (OPP1130226) and by a bilateral grant between the Research Foundation-Flanders (FWO-Vlaanderen) and the Vietnamese National Foundation for Science and Technology Development (NAFOSTED; G0D9318N).

## Supplementary Material

suppl_data_coab099
