# Corrigendum to: Drying banana seeds for *ex situ* conservation

**DOI:** 10.1093/conphys/coac005

**Published:** 2022-01-26

**Authors:** Simon Kallow, Manuela Garcia Zuluaga, Natalia Fanega Sleziak, Bayu Nugraha, Arne Mertens, Steven B Janssens, Lavernee Gueco, Michelle Lyka Valle-Descalsota, Tuong Dang Vu, Dang Toan Vu, Loan Thi Le, Filip Vandelook, John B Dickie, Pieter Verboven, Rony Swennen, Bart Panis

**Affiliations:** 1 Royal Botanic Gardens Kew, Millennium Seed Bank, Wakehurst, Ardingly, Sussex, RH17 6TN, UK; 2 Department of Biosystems, Katholieke Universiteit Leuven, Willem de Croylaan 42, 3001 Leuven, Belgium; 3 Meise Botanic Garden, Nieuwelaan 38, 1860 Meise, Belgium; 4 Agricultural and Biosystems Engineering Department, Universitas Gadjah Mada, Jl. Flora No. 1, Sleman, Yogyakarta 55281, Indonesia; 5 Department of Biology, Katholieke Universiteit Leuven, Kasteelpark Arenberg 31, 3001 Leuven, Belgium; 6 National Plant Genetic Resources Laboratory, Institute of Plant Breeding, College of Agriculture and Food Science, University of the Philippines, Los Baños, 4031 Laguna, Philippines; 7 Research Planning and International Cooperation Department, Plant Resources Center, VAAS, Ha Noi, Viet Nam; 8 Genebank Management Division, Plant Resources Center, VAAS, Ha Noi, Viet Nam; 9 International Institute of Tropical Agriculture, Plot 15B Naguru East Road, Upper Naguru, Box 7878, Kampala, Uganda; 10 Bioversity International, Willem de Croylaan 42, 3001 Leuven, Belgium

*Conserv Physiol* 10(1): coab099; doi: 10.1093/conphys/coab099

In the originally published version of this manuscript, the spelling of the co-author’s name was incorrect. The name should be “Loan Thi Le” instead of “Loan Thi Li”. This error has now been corrected online.

