# Supplementary material for: Drying banana seeds for ex situ conservation
Source: Conserv Physiol. 2022 Jan 12;10(1):coab099. doi: 10.1093/conphys/coab099 (PMC9041424; doi:10.1093/conphys/coab099)
Supplement: suppl_data_coab099 [file suppl_data_coab099.zip › DryingBananaSeeds_ConPhy_SuppFigsTables_compressed.docx]

# Supplementary Figures and Tables

| Bunch number | bunch | fruit | seed |
| --- | --- | --- | --- |
| 1 |  | 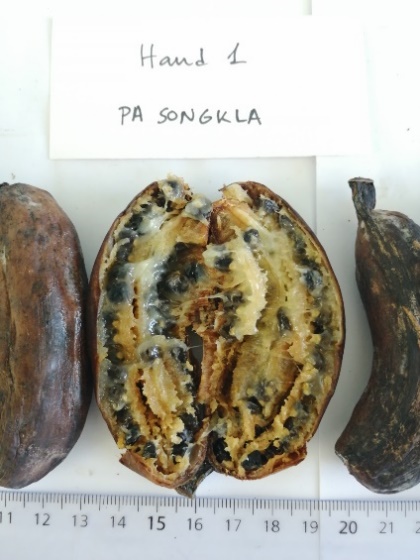 | 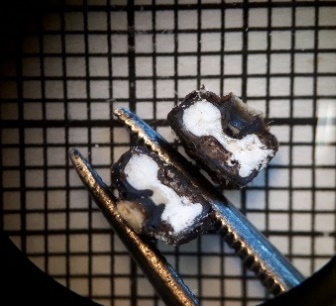 |
| 2 |  | 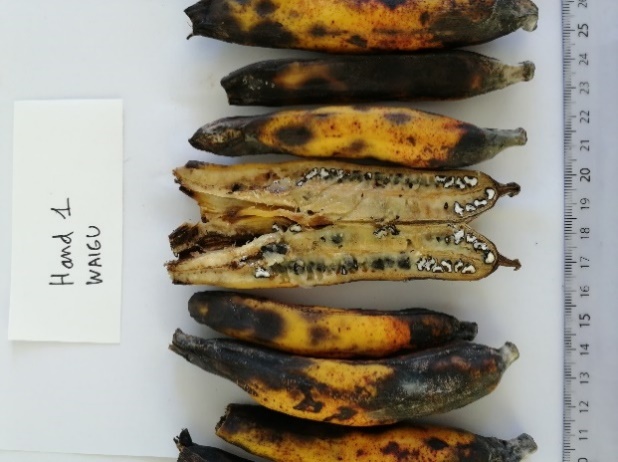 | 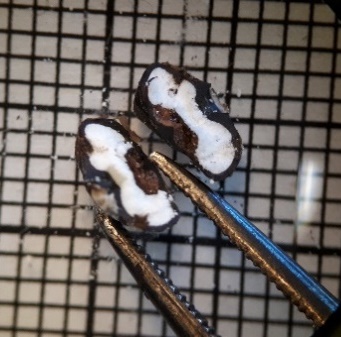 |
| 3 |  | 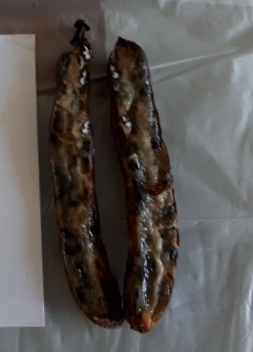 | 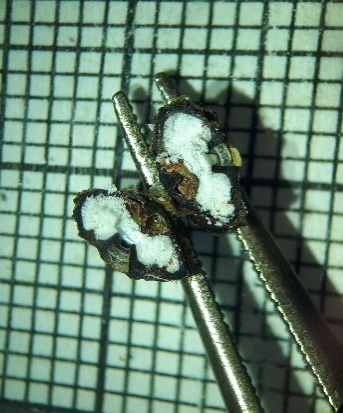 |
| 4 | 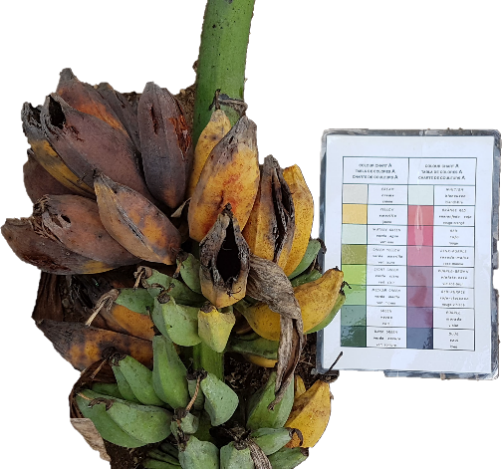 | 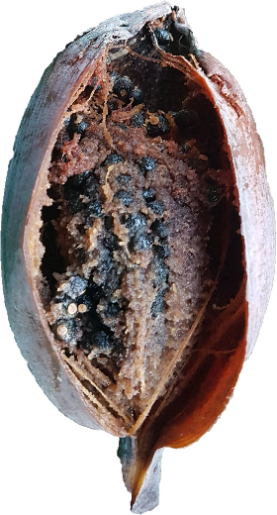 | 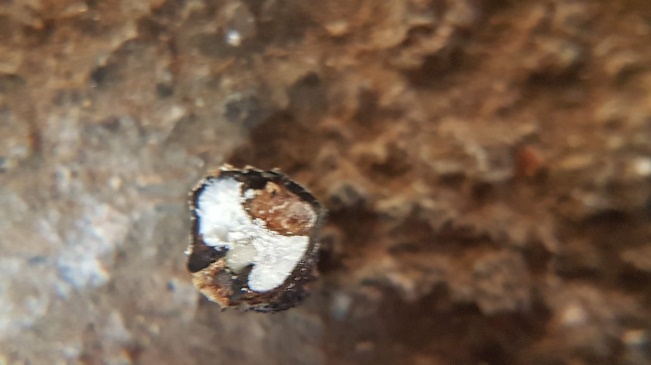 |
| 5 | 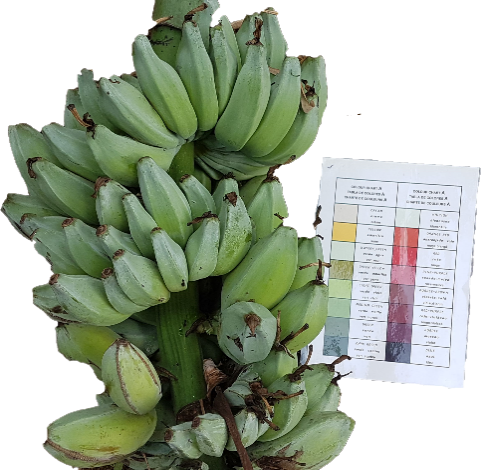 | 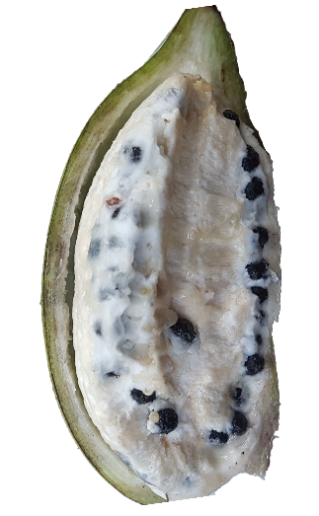 | 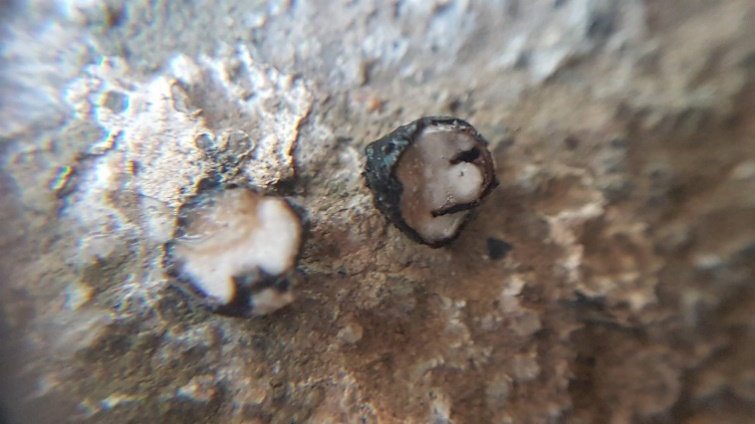 |
| 6 | 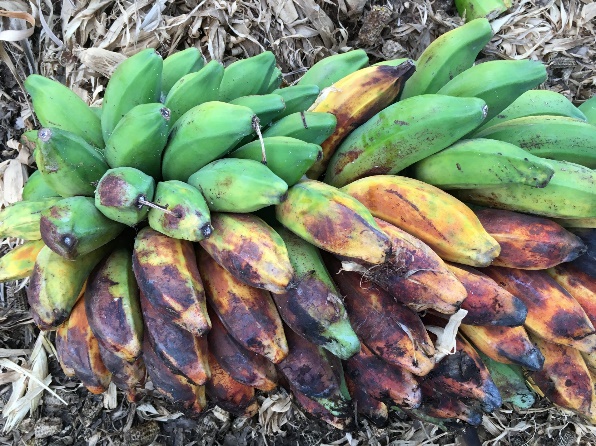 | 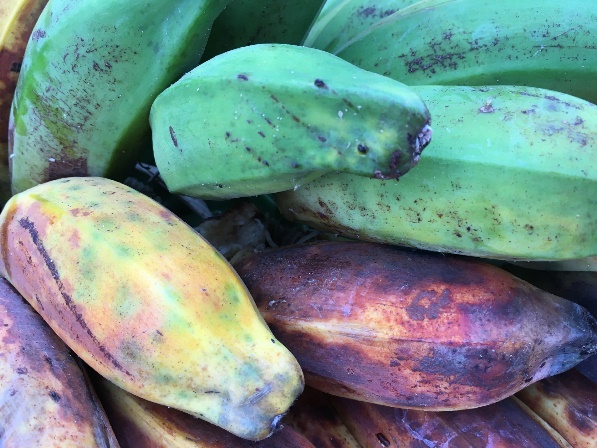 | 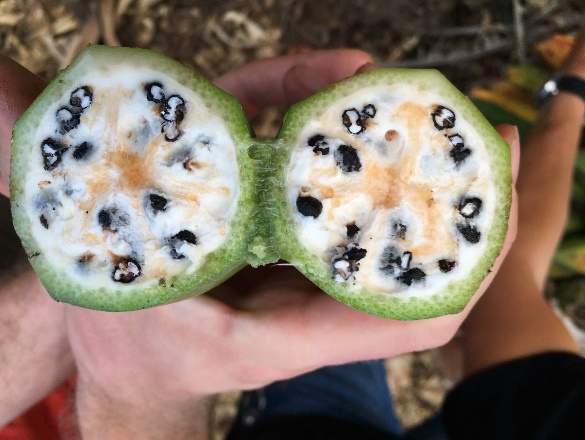 |
| 7 | 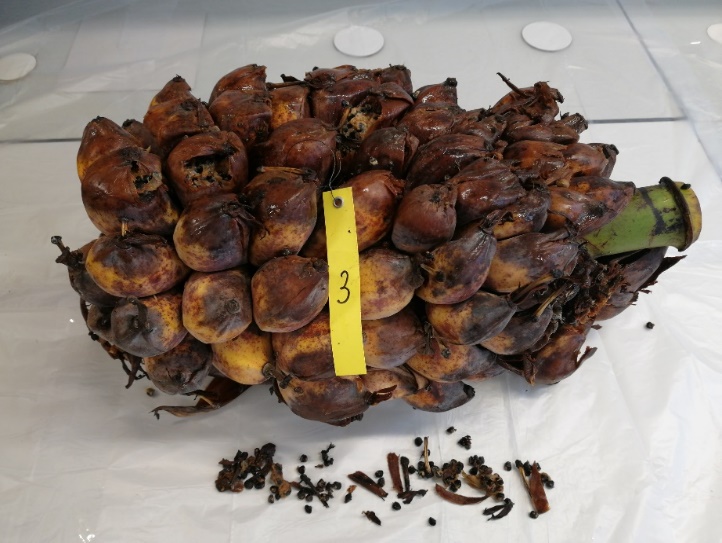 | 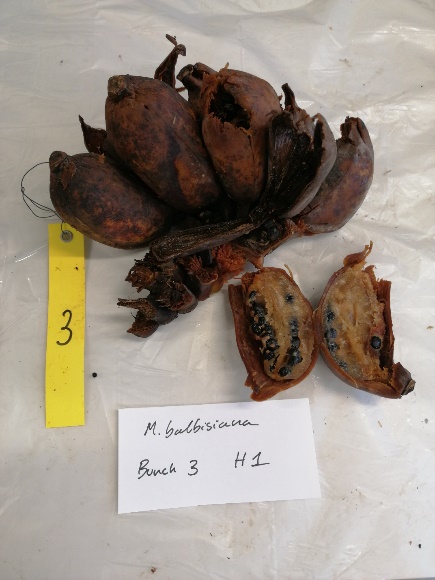 |  |
| 8 | 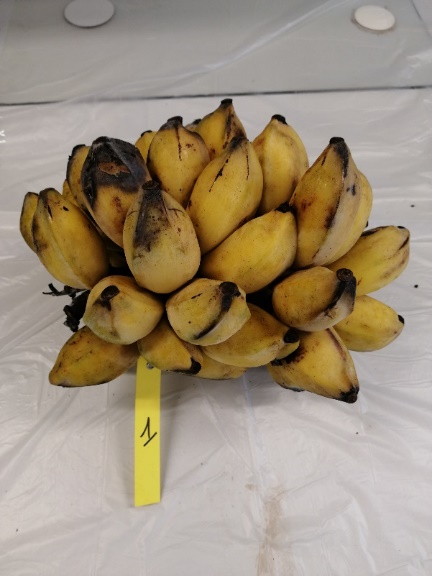 | 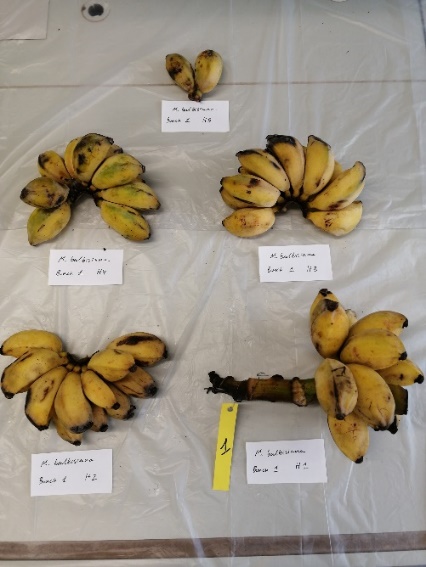 |  |
| 9* | 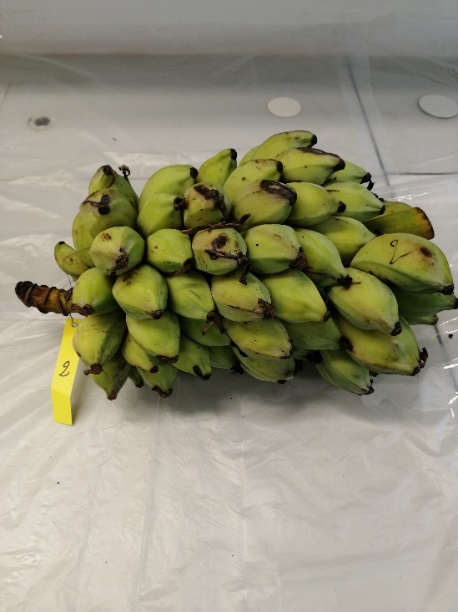 | 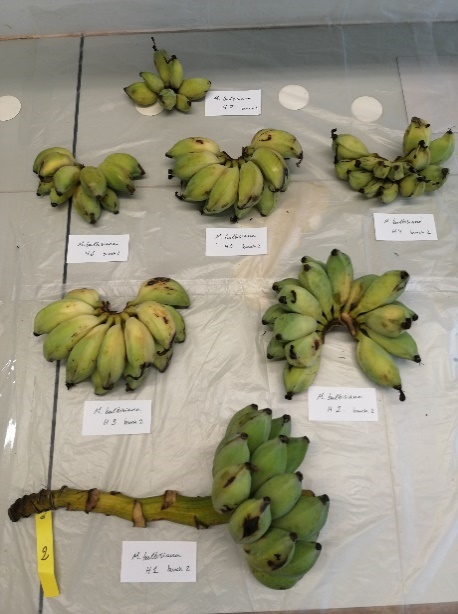 |  |

**Figure S1.** Photographs of bunches, fruit and seeds used in experiments, bunch numbers refer to those in Table 1; * not used in experiments as embryos were not developed enough.


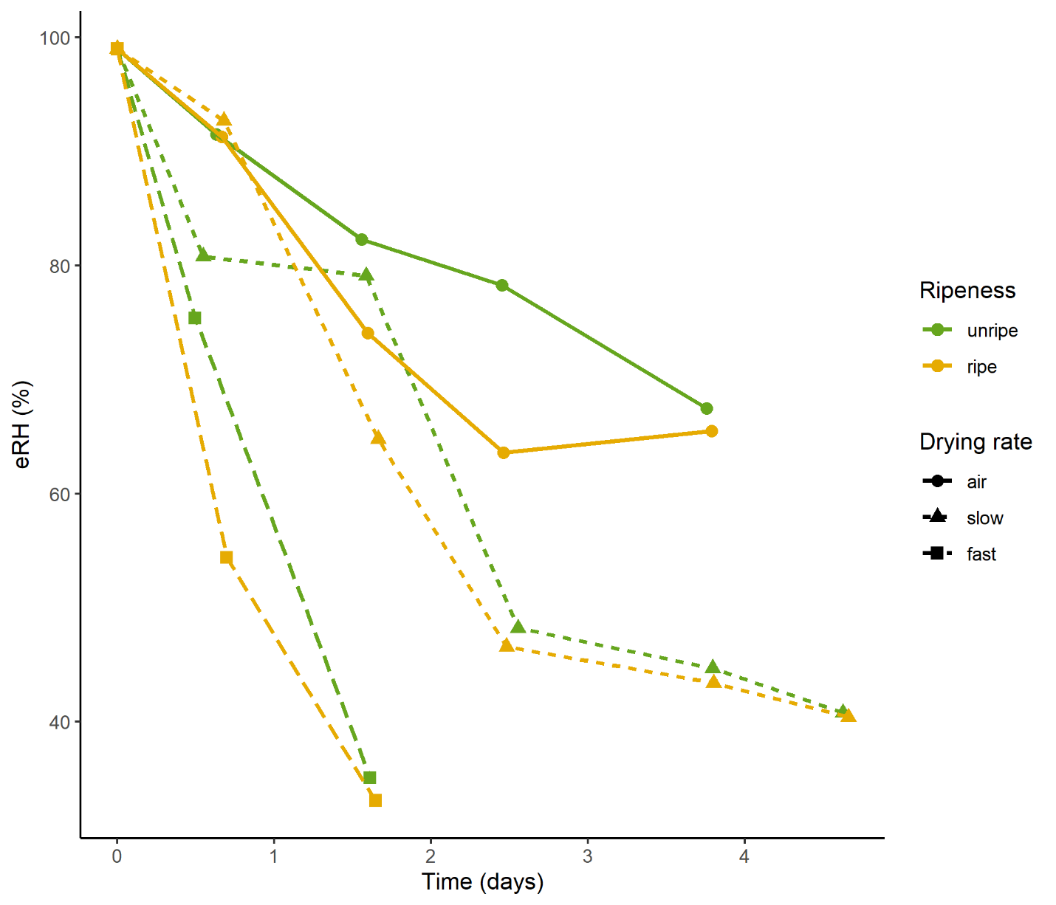


**Figure S2.** Drying rate of *M. balbisiana* seeds in Experiment 3, seeds taken from ripe and unripe fruit of the same bunch and dried in paper bags ‘air’, or with two levels of amount of silica gel ‘slow’ and ‘fast’.

**Table S1**. Lithium Chloride solutions used in experiment 1 to make 65ml of solution.

| LiCl (g) | RH (%) at 20°C | |  |
| --- | --- | --- | --- |
| 0 | 100 | |  |
| 17.88 | 65 | |  |
| 28.6 | 40 | |  |
| 41.6 | 20 | |  |
| 47.76 | 15 | |  |
| 56.55 | 11 | |  |
| Silica gel | 4 |  | |
