# Supplementary material for: Drying banana seeds for ex situ conservation
Source: Conserv Physiol. 2022 Jan 12;10(1):coab099. doi: 10.1093/conphys/coab099 (PMC9041424; doi:10.1093/conphys/coab099)
Supplement: suppl_data_coab099 [file suppl_data_coab099.zip › DryingBananaSeeds_ConPhy_SuppMethods.docx]

## Supplementary Methods

*Experiment 4: The effect of drying and imbibition on seed morphology: CT scanning and image analysis*

We developed a protocol to simultaneously CT scan eight seeds by arranging in two levels of four seeds, using two Styrofoam discs with a diameter of approximately 2 cm. Four indentations (3 mm diameter) were made with a small knife on each disc and a seed was placed in each. Discs were then stacked together and secured with tape. Samples of seeds were then scanned using a Phoenix Nanotom µ-CT system (General Electric, Heidelberg, Germany) on a 12-bit 2304 x 2304 detector. The scanner operated at a voltage of 80 kV and 240 µA current, without the use of a filter. The distance from the X-ray source to the sample measured 94.999 mm and to the detector 500 mm. These settings allowed for a 9.5 µm resolution and produced images with the least noise. For each sample, 1200 projection images were captured. The µ-CT projection images were reconstructed using Phoenix CT software (GE inspection Technology, Germany). This reconstruction created stacks of 8-bit grayscale images. Stacks were processed using Avizo 2019.3 (Thermo Fisher Scientific, Waltham, US). The scanof each seed was cropped and processed individually. A median filter was first applied to reduce image noise. Grayscale images were converted to binary images using the Auto Thresholding tool, with Auto threshold: High. Small spots up to 150 pixels around seeds were deleted using the Remove Small Spots tool. All leftover spots were removed manually. A transparent visualization of all the surfaces within the seed was computed with the Generate Surface tool followed by the Surface View tool, choosing color: transparent. The volume of the seed mass was measured using the Label Analysis tool, linking the intensity to the grayscale image and using a standard shape analysis. To measure the volume of entire seeds, air holes in binary images were filled with the Fill Holes tool. The Label Analysis tool was used to calculate the volume as before. Finally, to measure the volume of air inside seeds, the grayscale image was converted to a binary image using the Auto Thresholding tool but this time selecting Auto threshold: Low. The resulting binary image was the air inside the seed and surrounding the seed. This image was then masked with the image of the filled seed organ to produce a binary image with only the air inside the seed. Again, with the Label Analysis tool, the volume of the air inside the seed was measured. To segment the embryo, the grayscale images were converted to binary images using the Interactive Thresholding tool. The intensity range was changed such as to color as much of the embryo blue. A second set of binary images was then computed with the Fill Holes tool and subsequently eroded up to 60 pixels with the Erosion tool. These images were then masked on the first set of binary images to remove most of the seed that did not include the embryo. The Remove Small Spots tool was used to delete spots up to 5000 pixels that were not part of the embryo. Leftover spots were removed manually in the segmentation panel with the brush tool and by scrolling through all the slides to delete unwanted spots. The Label Analysis tool was used to measure the volume of the embryo.
